# Supplementary material for: Using Sandwiched Silicon/Reduced Graphene Oxide Composites with Dual Hybridization for Their Stable Lithium Storage Properties
Source: Molecules. 2024 May 7;29(10):2178. doi: 10.3390/molecules29102178 (PMC11124151; doi:10.3390/molecules29102178)
Supplement: Supplementary file 1 [file molecules-29-02178-s001.zip › molecules-2933007-supplementary.pdf]

Supplementary Material:

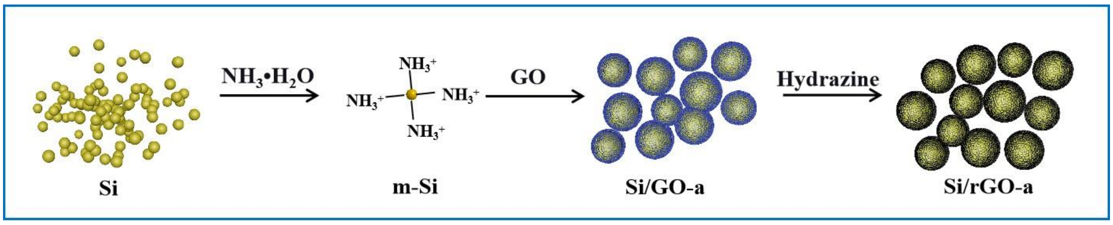

Figure S1 Schematic illustration of preparation of Si/rGO-a composite

Synthesis of Si/rGO-a composite: 200 mg of Si NPs and 100 ml of ethanol were mixed and dispersed ultrasonically for 2 h. Then, 50 ml deionized water and 2 ml ammonium hydroxide ($NH_3·H_2O$) were added into the reactor and treated ultrasonically for 0.5 h. Then added 250 ml GO suspension (1 mg/ml) and magnetically stirred for 6 h to obtain homogenous Si/GO-a. Subsequently, by adding 250 μl of $N_2H_4·H_2O$, reactions were carried out for 12 h in a water bath of 85 ℃. The product was then filtered with an organic filter membrane, washed three times with deionized water, and dried in vacuum for 10 h at 60 ℃ to obtain the Si/rGO-a composite.

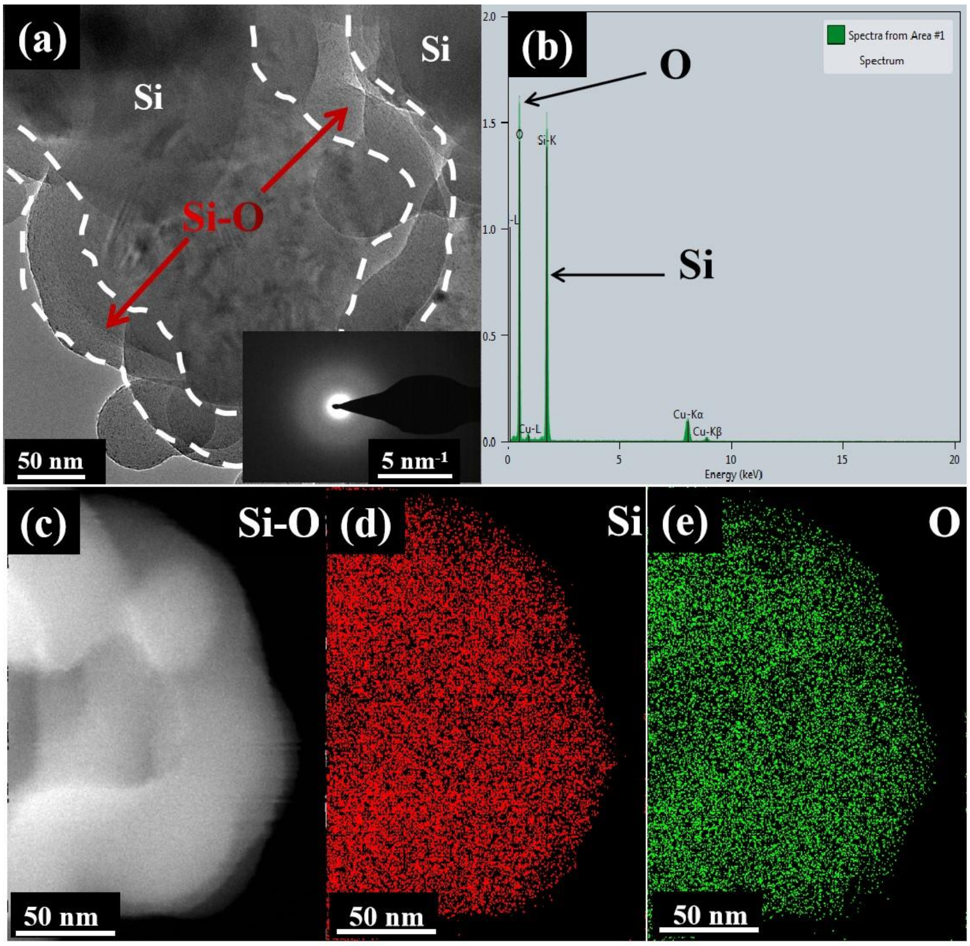

Figure S2 Morphology of Si-O on the surface of m-Si. (a) TEM image of Si-O. Insets was diffraction pattern of Si-O. (b) EDS analysis of Si-O. (c) HAADF - STEM image of Si-O. (d, e) Distribution of Si and O elements in mapping mode.

Using TEM to further demonstrate that ammonia water can react with Si. According to chemical reactions (1) (2) and (3), Si reacts with $NH_3 \cdot H_2O$ to generate $(NH_4)_2SiO_3$. During the preparation of TEM samples, such as a chemical reaction (4) occurs, and the $(NH_4)_2SiO_3$ decomposes to form silicon oxide (Labeled Si-O). Fig. S4a shows the surface oxide Si-O of m-Si, and the diffraction pattern in the inset shows no diffraction spots, indicating that the oxide is an amorphous structure. The EDS analysis in Fig.S4b shows that the oxide is composed of Si and O elements. Fig. S4c is a HAADF - STEM image of the Si-O. Fig. S4d and Fig. S4e show the elemental mappings of the Si-O, revealing the distribution of Si and O in the Si-O. Fig.S5a is the

XPS spectrum of Si-O, and the O1s curve in the inset shows no Si-Si bonds. TEM and XPS characterization indicate that the surface silicon element of Si particles reacts with $NH_3 \cdot H_2O$ to form $(NH_4)_2SiO_3$, and the m-Si surface is connected with amino groups.

$$NH_3 \cdot H_2O \rightarrow NH_4^+ + OH^- \quad (1)$$

$$4OH^- + Si \rightarrow H_4SiO_4 \rightarrow H_2SiO_3 + H_2O \quad (2)$$

$$4NH_4^+ + H_2SiO_3 \rightarrow (NH_4)_2SiO_3 \quad (3)$$

$$(NH_4)_2SiO_3 \rightarrow NH_3 \cdot H_2O + SiO_2 \quad (4)$$

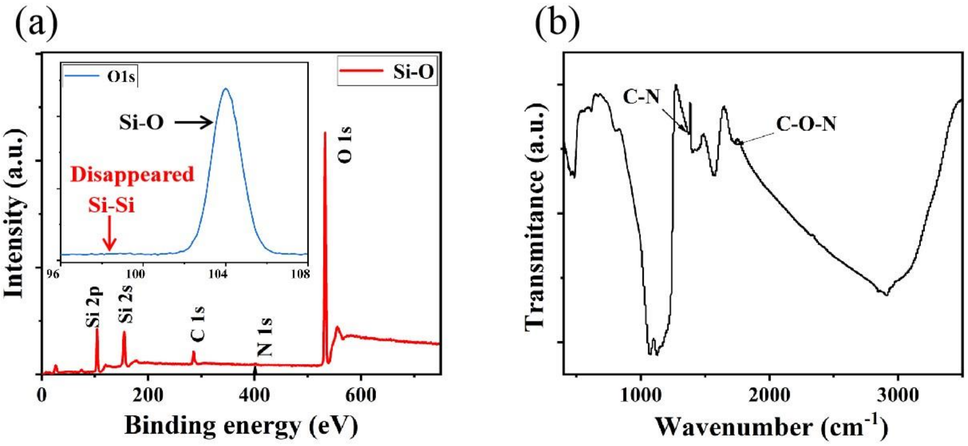

Figure S3 (a) XPS spectra of Si-O, inset is the Si2p spctra of Si-O. (b) FI-IR spectra of Si/GO.

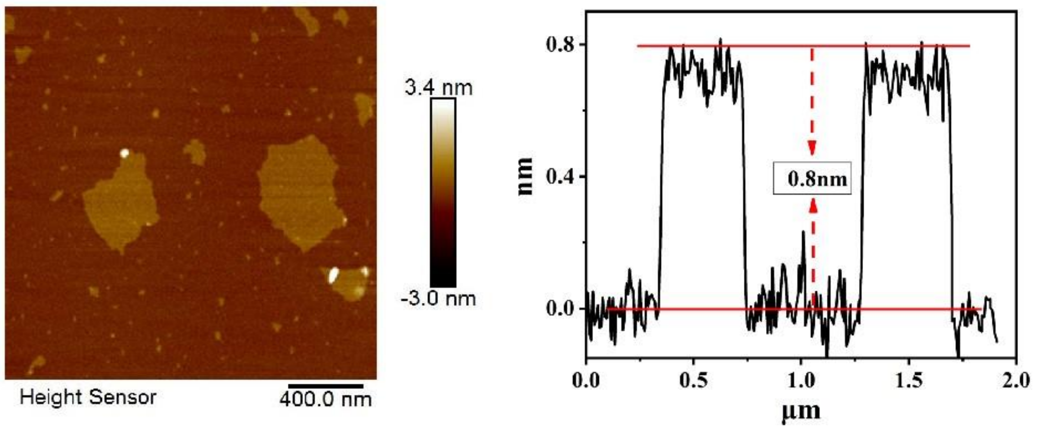

Figure S4 Tapping mode AFM image and height profile of a single layer of GO.

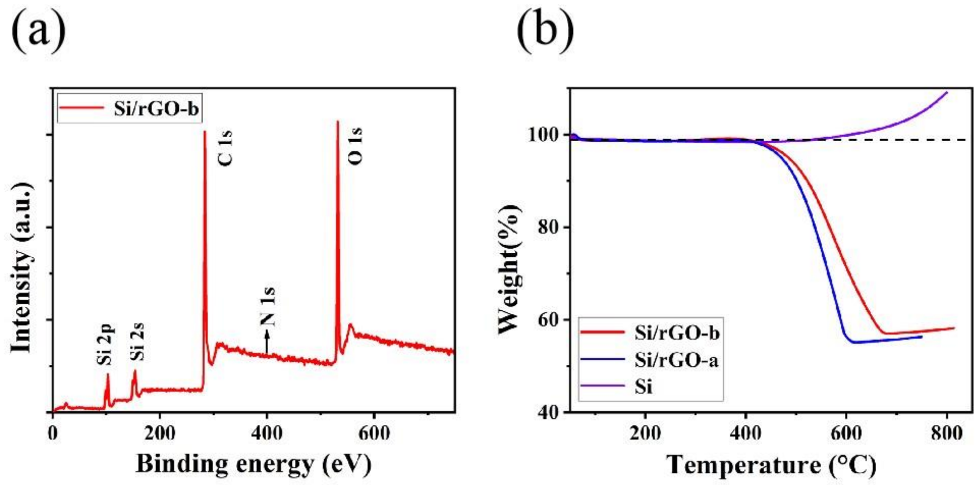

Figure S5 (a) XPS spectra of Si/rGO-b. (b) TG plots of Si/rGO-b, Si/rGO-a and pure Si.

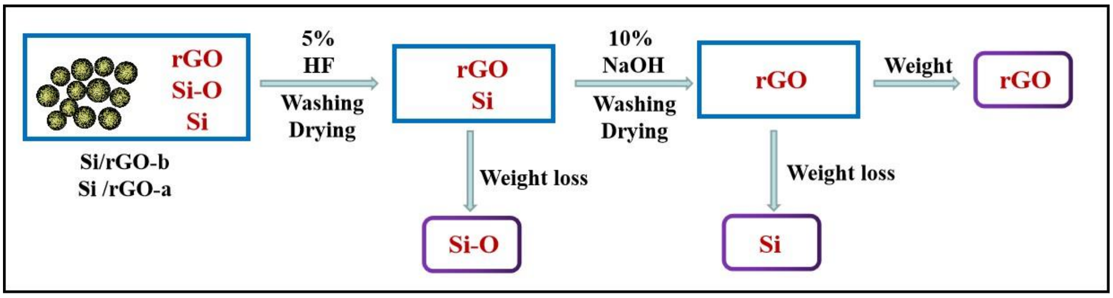

Figure S6 Schematic diagram of component analysis for Si/rGO-b and Si/rGO-a composites.

Composition analysis of composite: SiO can react chemically with HF acid, while Si reacts chemically with NaOH. Use the subtraction method to determine the various components of composite materials. React with the composite material using a 5% HF solution, dry the remaining solid and weigh it. The weight loss is the content of SiO. Then, a 10% NaOH solution is used to react with the remaining solid, resulting in a weight loss of pure Si content, and the final remaining weight is the content of rGO.

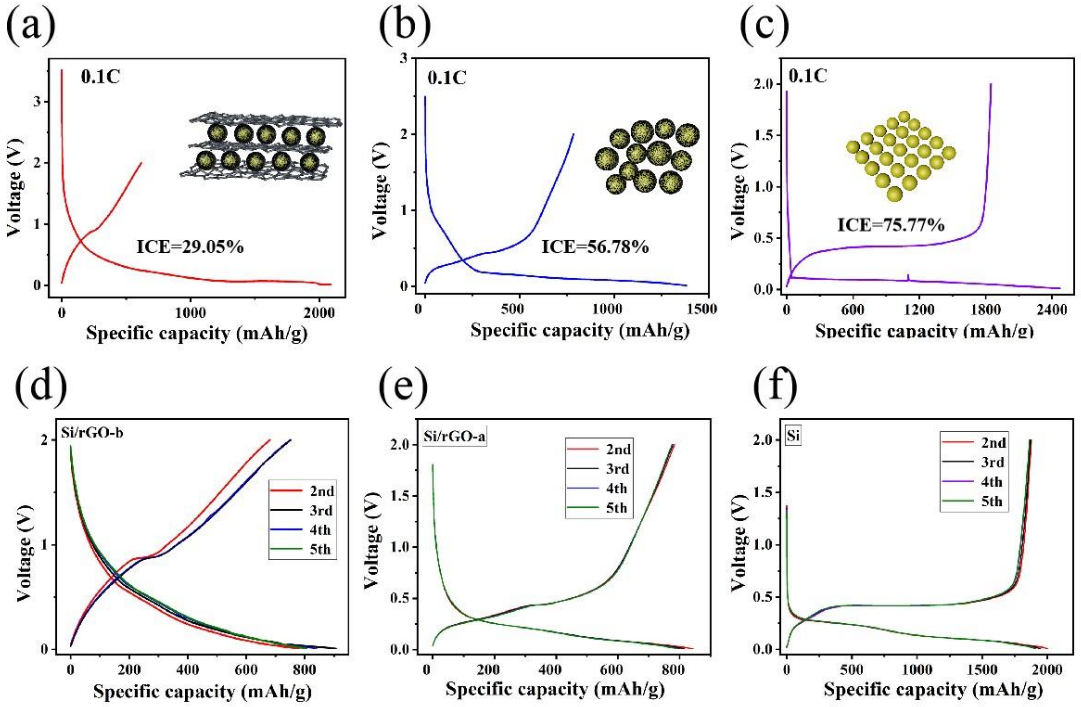

Figure S7. (a - c) Initial discharge/charge curves of Si/rGO-b, Si/rGO-a and Si electrode at current density of 0.1 A/g. (d - f) Corresponding curves for 2nd - 5th cycle.

Table S1 Comparison of Li storage in Si/rGO-b and reported Si-based anodes.

Electrode	Voltage(V)	Current density (mA /g)	Cycle number	Cycling stability (mA h/g)	Reference
Si–graphite/C	0.005 - 1.0	200	100	517	[1]
Si/graphite/graphene	0.005 - 2.0	372	300	445	[2]
Si/porous–C/N–graphene	0.005 - 2.0	2000	110	1168.5	[3]
porous Si/C	0.005 - 1.5	87	450	650	[4]
C/Si/rGO	0.005 - 1.0	2000	350	800	[5]
3D Si/N–graphite/C	0.005 - 1.5	200	400	900	[6]
Si/graphene	0.005 - 3.0	500	300	910	[7]
Si/rGO	0.005 - 3.0	500	300	1200	[8]
Si/rGO	0.005 - 1.5	1000	600	483	[9]
Si/rGO	0.005 - 1.5	1000	800	511	This work

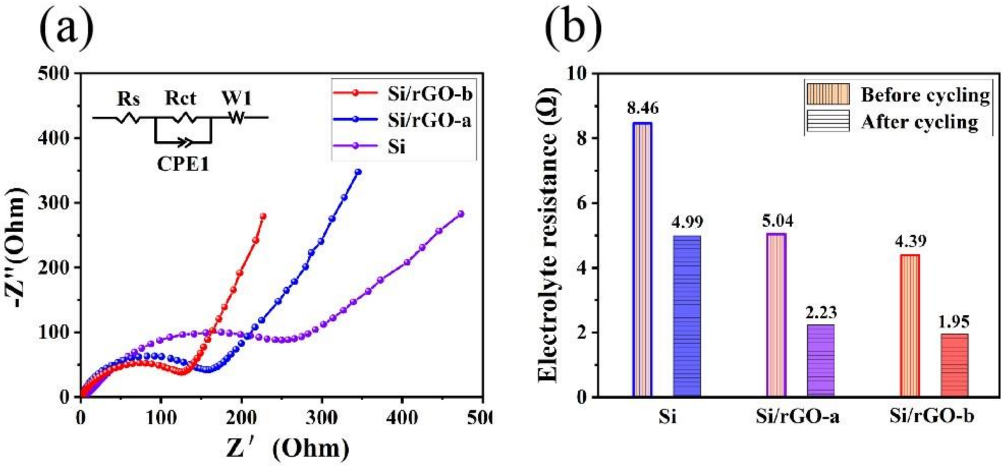

Figure S8. (a) Initial Nyquist plot of Si/rGO-b Si/rGO-a and Si electrodes. Inset shows equivalent circuit for the electrodes. (b) R_s of Si/rGO-b Si/rGO-a and Si electrodes before and after 50 cycling.

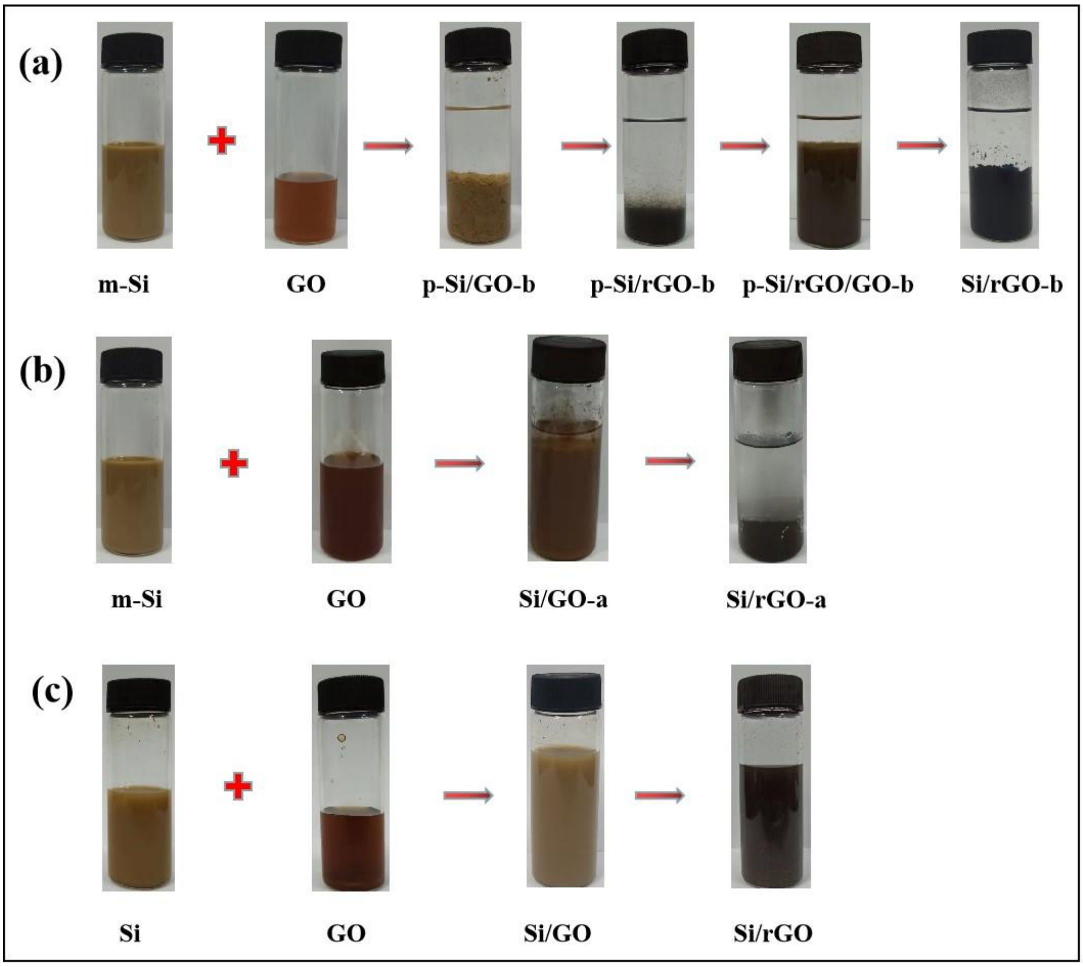

Figure S9 (a, b, c) The digital photo of the preparation of Si/rGO-b, Si/rGO-a, Si/rGO composite.

The self-assembly of m-Si and GO forms p-Si/GO-b, indicating that the surface of Si is connected to amino groups and exhibits positive charge. In the reaction system for synthesizing p-Si/rGO-b and Si/rGO-b, the separation of solid products from solvent liquids indicates that rGO is coated on the surface of Si particles without free rGO. The experimental phenomenon of m-Si and GO synthesizing Si/rGO-a is similar to the synthesis phenomenon of Si/rGO-b. By combining pure Si with GO, the Si particles are not charged and cannot self-assemble with negatively charged GO. Therefore, the solid and solvent liquid in the system are not separated. There is a large amount of free rGO present in the final Si/rGO turbidity.

Table S2 Statistics of main reagents in the experiment.

Materials	Purity	Manufacturer
Si	99.99%, 80 - 100 nm	Dongguan Kelude Experimental Equipment Technology Co., Ltd.
Graphite	99.5%, 10 - 15 μm	China National Medicines Corporation Ltd.
H_2SO_4	95%	China National Medicines Corporation Ltd.
$KMnO_4$	≥99.5%	China National Medicines Corporation Ltd.
$NaNO_3$	≥99.5	China National Medicines Corporation Ltd.
HCl	36 - 38%	China National Medicines Corporation Ltd.
H_2O_2	30%	China National Medicines Corporation Ltd.
Ethanol	99.99%	China National Medicines Corporation Ltd.
$NH_3 \cdot H_2O$	28% - 30%	China National Medicines Corporation Ltd.
$N_2H_4 \cdot H_2O$	85%	China National Medicines Corporation Ltd
